# Gratitude and happiness among university students: a cross-sectional study in teacher education

**DOI:** 10.3389/fpsyg.2026.1974252

**Published:** 2026-10-15

**Authors:** Hernán Vicente Rojas-Moscoso, Adrián Orlando Manrique-Rojas, Naylea Nayeli Velasquez Cutipa, Pilar Valeria Zapana-Aguilar, Kevin Mario Laura-De La Cruz

**Affiliations:** 1 Universidad Nacional Jorge Basadre Grohmann, Tacna, Peru; 2 Universidad Católica Santa María, Arequipa, Peru

**Keywords:** bootstrap confidence intervals, gratitude, happiness, robust regression, university students

## Abstract

**Introduction:**

This study examined the association between gratitude and happiness among education students at a public university in Tacna, Peru, including four dimensions of happiness: positive sense of life, life satisfaction, personal fulfillment, and joy of living.

**Methods:**

A quantitative, non-experimental, cross-sectional correlational design was used. Participants were 507 students (313 women and 194 men) aged 18–37 years. Gratitude and happiness were assessed using the Lima Gratitude Scale and the Lima Happiness Scale. Internal consistency and associations were examined using Cronbach's alpha, model-based omega, Spearman's rho, bootstrap confidence intervals, and Holm-adjusted *p*-values.

**Results:**

Internal consistency was good for gratitude (α = 0.807, 95% bootstrap CI [0.777, 0.831]; ω = 0.823) and excellent for happiness (α = 0.942, 95% bootstrap CI [0.933, 0.950]; ω = 0.943). Gratitude was positively associated with overall happiness, Spearman's ρ = 0.447, 95% bootstrap CI [0.368, 0.519], *p* < 0.001. Dimension-specific correlations ranged from ρ = 0.351 for personal fulfillment to ρ = 0.420 for joy of living; all remained significant after Holm adjustment. Excluding the participant with one reconstructed item response produced a similar overall correlation (ρ = 0.448).

**Discussion:**

Gratitude showed a robust, moderately sized positive association with happiness and its four dimensions among teacher education students. However, the cross-sectional design precludes causal inference, and the single-institution convenience sample limits generalizability. Longitudinal and multi-institutional studies are needed to determine the temporal direction and broader applicability of these associations.

## 1 Introduction

Teacher education combines academic study with preparation for relationships and responsibilities in educational settings. Understanding students' wellbeing during this preparation is relevant both to their own university experience and to the support available to prospective teachers. Research on coping and psychological distress in this population indicates that adjustment cannot be understood solely in terms of academic performance (Gustems-Carnicer and Calderón, 2013).

Gratitude and happiness offer complementary perspectives on this experience. Gratitude concerns recognizing and valuing benefits received, whereas happiness concerns the individual's positive evaluation and experience of life (Alarcón, 2006, 2014). Their association may help identify psychological resources relevant to teacher preparation, while leaving open how educational demands and social conditions shape both constructs.

Teacher education provides a substantively relevant setting for studying these resources. Prospective teachers are preparing to support children's and adolescents' learning and development, respond to diverse support needs, and manage relationships in emotionally demanding classrooms. Their preparation therefore involves both their own adjustment to university and the development of capacities relevant to future educational relationships. Research on coping and psychological distress among teacher education students, together with studies of wellbeing interventions in this population, supports examining these demands during initial preparation (Gustems-Carnicer and Calderón, 2013; Böke et al., 2024). Gratitude may be relevant because recognizing support and responding to others' contributions could help sustain the relationships on which teacher learning depends; this proposed mechanism requires empirical testing.

Positive psychology regards gratitude as one of the character strengths that promote optimal human functioning. Recently, in a 2-week daily diary study, Wagner and Gander (2025) found that gratitude is among the strengths showing the greatest convergence between a stable trait and its daily expression; in other words, it is among the strengths that remain most consistent across time and situations, and this consistency is associated with greater emotional wellbeing. In its emotional dimension, being grateful involves recognizing positive events and expressing appreciation to those who make them possible.

This ability to recognize benefits received also serves a social function because it directs individuals toward reciprocity and strengthens emotional bonds with others. Applying the broaden-and-build theory of positive emotions to a sample of Chinese volunteers, Yang et al. (2023) found that gratitude was associated with subjective wellbeing and continued volunteer engagement through indirect pathways linked to prosocial motivation. This finding is consistent with the proposed role of positive emotions in psychological and social resources, although the reported associations do not establish causality.

In educational settings, Zhang et al. (2022), studying 801 pre-service teachers in China, reported associations linking gratitude with career goal self-efficacy through meaning in life and career calling. Their questionnaire-based mediation model identifies plausible pathways, without establishing their causal direction. Similarly, recent interventions show that expressing gratitude, together with performing acts of kindness and deliberately applying character strengths, improves university students' subjective wellbeing (Ekwonye et al., 2026). A meta-analysis of randomized controlled trials also confirms that gratitude interventions have a significant, albeit small-to-moderate, effect on life satisfaction, positive affect, and happiness (Kirca et al., 2023).

Research on student wellbeing includes the study of positive experiences as well as psychological difficulties. A recent systematic review of research conducted in Latin America found that most studies of happiness among university students use correlational designs and that optimism and interpersonal relationships are among the factors most strongly associated with this construct (Rojas-Solís et al., 2022).

(Alarcón, 2009, p. 137) defines happiness as “a more or less enduring state of satisfaction subjectively experienced by an individual who possesses a desired good.” Several characteristics follow from this definition: happiness is a subjective feeling; it may be enduring but is not permanent; it depends on possessing the desired good; it may arise from multiple sources (material, ethical, or psychological); and it varies according to cultural context.

Based on a factor analysis conducted with Peruvian university students, Alarcón (2006) identified four dimensions of happiness: (a) positive sense of life, referring to the relative absence of depression, pessimism, and uneasiness; (b) life satisfaction, associated with the perception of achieving one's own goals; (c) personal fulfillment, related to emotional tranquility, self-sufficiency, and the pursuit of meaningful goals; and (d) joy of living, which refers to feeling positive about one's life experiences.

Research specifically addressing teacher preparation places wellbeing within a broader educational context. Haldimann et al. (2024) examined the construct validity of pre-service teacher wellbeing across three countries, drawing attention to the need for appropriate measurement across settings. Among 98 teacher education students, Gustems-Carnicer and Calderón (2013) linked approach-oriented coping with lower psychological distress, whereas avoidance was associated with poorer psychological functioning. In Spain, Huertas-Delgado et al. (2020) examined life satisfaction and personal values in 565 teacher education students, demonstrating the relevance of studying students' evaluative wellbeing alongside their value orientations. These studies address related but distinct aspects of students' experience; they should not be interpreted as interchangeable measures of happiness.

Intervention research also suggests that wellbeing during teacher preparation depends on how learning and support are organized. In Hong Kong, Datu et al. (2023) randomly assigned 128 pre-service preschool teachers to different delivery formats of a multicomponent positive psychology intervention or a wait-list control condition. The online workshop produced the strongest improvements in several wellbeing outcomes, whereas the smartphone-only condition did not significantly improve psychological outcomes. In France, Legrain et al. (2023) investigated perceived quality of life through motivational and instructional indicators among 69 pre-service physical education teachers; the benefits of cooperative training differed by outcome and training condition. Together, these findings support examining individual psychological resources in relation to educational conditions, rather than assuming that a positive disposition is sufficient to ensure wellbeing.

In the present study, happiness refers to the affective and evaluative construct operationalized by the Lima Happiness Scale. It is relevant to the broader study of student wellbeing, but it is not a diagnostic measure of mental health or a comprehensive assessment of quality of life. This distinction allows the findings to inform that literature without equating higher happiness with the absence of psychological symptoms or with better functioning in every area of life.

Recent evidence supports a positive relationship between gratitude and happiness or subjective wellbeing. In a positive psychology intervention program for engineering students, Kalamatianos et al. (2023) found a strong relationship between gratitude and subjective happiness (*r* = 0.69), as well as optimism. Scurtu-Tura et al. (2024) found that gratitude emotions partially mediate the relationships of subjective happiness and positive affect with life satisfaction among Spanish adults, with a somewhat stronger association in women than in men. Chauhan et al. (2025) reported an association between gratitude and life satisfaction, with mental wellbeing as a statistical mediator. In a systematic review, Kerry et al. (2023) concluded that gratitude and life satisfaction show a substantial positive correlation, with effects replicated across different populations and cultural contexts. In university workplaces, Garg et al. (2022) reported relationships between gratitude and workplace happiness among university teachers, with psychological and social capital as proposed mediating variables.

Against this background, and given the relative scarcity of studies examining the practical magnitude, not only the statistical significance, of the relationship between gratitude and specific dimensions of happiness among education students, the present study focused on students preparing for teaching careers and aimed to determine the relationship between gratitude and happiness within the Professional School of Education of a public university in Tacna. The target population was therefore this teacher education setting, rather than the university student population as a whole. Specifically, it sought to determine the relationship between gratitude and each of the four dimensions of happiness proposed by Alarcón (2006): positive sense of life, life satisfaction, personal fulfillment, and joy of living.

## 2 Methods

### 2.1 Design

The study employed a quantitative approach with a non-experimental, cross-sectional, correlational design (Hernández Sampieri and Mendoza Torres, 2018; Supo, 2020). Data were collected during the second semester of the 2023 academic year.

### 2.2 Participants

The population comprised students enrolled from the first through the fifth year in five degree programs offered by the Professional School of Education (ESED) at Universidad Nacional Jorge Basadre Grohmann, a public university in Tacna, Peru. Non-probability convenience sampling was used: students who were present during classroom administration and voluntarily agreed to participate were recruited. The sample comprised 507 participants: 313 women (61.7%) and 194 men (38.3%), aged 18–37 years. The year-of-study distribution was 114 first-year, 126 second-year, 112 third-year, 66 fourth-year, and 89 fifth-year students. Only enrolled students who voluntarily participated were included.

The focus on this school was justified by the relevance of psychological resources to preparation for educational roles, as outlined in the Introduction. Including students across five programs and all five academic years allowed the association to be examined across different stages and specializations within that setting. The inclusion of these programs and academic years does not establish representativeness, either within the institution or across other faculties and universities, because recruitment depended on attendance and willingness to participate. No comparison group from non-teaching degree programs was included.

### 2.3 Instruments

#### 2.3.1 Lima Gratitude Scale

Gratitude was assessed using the Lima Gratitude Scale (LGS; Alarcón, 2014), which comprises 18 Likert-type items with five response options (1 = strongly disagree, 2 = disagree, 3 = neither agree nor disagree, 4 = agree, and 5 = strongly agree, before reverse scoring) grouped into three factors: reciprocity (nine items), moral obligation (seven items), and sentimental quality (two items). Total scores range from 18 to 90; higher scores indicate greater gratitude. Analyses reported item averages on the 1–5 metric rather than categories derived from cutoffs. In the original version, Alarcón (2014) reported very high internal consistency (alpha = 0.903; standardized-item alpha = 0.912) and equally high split-half coefficients (Spearman-Brown = 0.869; Guttman split-half = 0.869). Construct validity was supported by a Varimax-rotated factor analysis, with factor loadings ranging from 0.48 to 0.78.

Illustrative items from the original Spanish instrument are presented here in English translation: reciprocity, item 9, “I consider myself a grateful person”; moral obligation, item 4, “Acknowledging what we have received is a moral obligation”; and sentimental quality, item 6, “Gratitude ends when favors are repaid” (Alarcón, 2014). Item 6 is negatively keyed: disagreement indicates greater gratitude. In the original scale, items 5, 6, and 11 require reverse scoring; the analysis used direction-scored responses as described below.

In the present sample, the LGS showed good internal consistency, α = 0.807, 95% bootstrap CI [0.777, 0.831], and model-based ω = 0.823. Reliability varied across dimensions: reciprocity α = 0.770, moral obligation α = 0.683, and sentimental quality α = 0.628.

#### 2.3.2 Lima Happiness Scale

Happiness was assessed using the Lima Happiness Scale (LHS; Alarcón, 2006), which comprises 27 Likert-type items with five response options (1 = strongly disagree, 2 = disagree, 3 = neither agree nor disagree, 4 = agree, and 5 = strongly agree, before reverse scoring) grouped into four factors: positive sense of life (11 items), life satisfaction (6 items), personal fulfillment (6 items), and joy of living (4 items). During its original development, Alarcón (2006) reported highly significant item-total correlations (*p* < 0.001; mean *r* = 0.511), high internal consistency (alpha = 0.916; Spearman-Brown = 0.884; Guttman = 0.882), and evidence of convergent and discriminant validity through its associations with the Eysenck Personality Inventory (*r* = 0.378 with extraversion; *r* = −0.450 with neuroticism) and measures of positive affect (*r* = 0.48) and negative affect (*r* = −0.51).

Illustrative items from the original Spanish instrument are presented here in English translation: positive sense of life, item 2, “I feel that my life is empty”; life satisfaction, item 4, “I am satisfied with my life”; personal fulfillment, item 24, “I consider myself fulfilled”; and joy of living, item 12, “Living is wonderful” (Alarcón, 2006). Item 2 is negatively keyed, so disagreement contributes to a higher happiness score. Total scores range from 27 to 135. After direction scoring, higher item, dimension, and total scores indicate greater happiness; analyses reported item averages on the 1–5 metric.

In the present sample, the LHS showed excellent internal consistency, α = 0.942, 95% bootstrap CI [0.933, 0.950], and model-based ω = 0.943. Dimension-level α values ranged from 0.799 for personal fulfillment to 0.902 for positive sense of life.

### 2.4 Procedure

The Dean of the Faculty of Education, Communication and Humanities, Universidad Nacional Jorge Basadre Grohmann was asked for and given permission to apply the instruments. Participation is optional and replies are confidential. All participants were 18 years or older and gave written informed permission before to completion of the surveys. Spanish-language scales were given on paper and scored using the original scoring keys. Each questionnaire took roughly 15 mins to complete, for a total of approximately 30 mins for both scales. There was no pay nor any other inducements. Simultaneous data were gathered during class hours on 20 days in the second semester of the 2023 academic year. The responses were entered in Microsoft Excel for statistical analysis.

### 2.5 Data analysis

The analysis used the item-level database and retained the original source sheet unchanged. Negatively keyed items were treated as already direction-scored in the supplied dataset so that higher values consistently indicated more gratitude or happiness; no second reversal was applied. All total and dimension scores were recalculated from the item responses. Overall item means were calculated across all items in each scale rather than as unweighted averages of dimension means. One blank F5 response was reconstructed as 4 because the archived happiness total and life-satisfaction subscore uniquely implied that value; excluding this participant yielded an essentially identical overall correlation (ρ = 0.448, *N* = 506, vs. ρ = 0.447, *N* = 507, with the reconstructed value included).

Initial data processing was performed in SPSS version 24.0 (IBM Corp., Armonk, NY, United States). The subsequent analyses reported here were conducted in Python (Python Software Foundation, Wilmington, DE, United States) using pandas, SciPy, and scikit-learn. Descriptive statistics included means, standard deviations, 95% confidence intervals, medians, interquartile ranges, skewness, and excess kurtosis. Internal consistency was evaluated with Cronbach's α, 3,000-resample bootstrap confidence intervals, and one-factor model-based ω; ω was not estimated for the two-item sentimental-quality dimension. Associations were estimated with Spearman's ρ and 5,000-resample bootstrap confidence intervals. Holm's procedure (Holm, 1979) controlled the familywise error rate across five correlations. A supplementary unadjusted ordinary least-squares model used standardized gratitude and happiness scores and HC3 robust standard errors. This model assessed the unadjusted linear association and did not control for potential confounders.

Distributional shape was summarized using skewness; skewness alone was not treated as evidence of normality. Spearman's rank correlation estimates monotonic associations without requiring normally distributed scores. The supplementary regression used HC3 standard errors to reduce sensitivity of inference to heteroskedasticity; this correction does not, by itself, establish model adequacy or address unmeasured confounding.

## 3 Results

### 3.1 Data quality and sample characteristics

The database contained 507 unique participants and no duplicated item-response profiles. All gratitude responses were complete. One happiness response was blank (F5 for participant 4) and was reconstructed as 4 from the internally stored total and dimension scores, leaving no missing item data. All item values were within the expected 1–5 range. Participants' descriptive characteristics are summarized in Table 1.

**Table 1:** Participants' descriptive characteristics.

Characteristic	*n*	% or summary
Total sample	507	100.0%
Age, years	507	Range 18–37
Women	313	61.7%
Men	194	38.3%
First year	114	22.5%
Second year	126	24.9%
Third year	112	22.1%
Fourth year	66	13.0%
Fifth year	89	17.6%
Program CNEA	92	18.1%
Program IDEX	90	17.8%
Program LELI	122	24.1%
Program MACI	81	16.0%
Program SPRO	122	24.1%

CNEA, Natural Sciences and Environmental Education; IDEX, Foreign Languages; LELI, Language and Literature; MACI, Mathematics, Computing, and Informatics; SPRO, Social Sciences and Sociocultural Promotion.

### 3.2 Reliability and descriptive statistics

The total gratitude scale showed good internal consistency (α = 0.807, 95% bootstrap CI [0.777, 0.831]; ω = 0.823), whereas the total happiness scale showed excellent internal consistency (α = 0.942, 95% bootstrap CI [0.933, 0.950]; ω = 0.943). Dimension reliability was strongest for positive sense of life (α = 0.902) and weakest for the two-item sentimental-quality dimension (α = 0.628), so interpretations of the latter should be cautious.

Corrected item–total correlations ranged from 0.193 to 0.573 for gratitude and from 0.490 to 0.720 for happiness. Gratitude item 11 showed the weakest item–total correlation (0.193); deleting it would increase α only slightly, from 0.807 to 0.812. The full 18-item scale was retained. These correlations describe item discrimination within each scale; they do not by themselves demonstrate discriminant validity between constructs.

As shown in Table 2 and Figure 1, the recalculated overall gratitude mean was 3.976 (SD = 0.438) on the 1–5 item metric. Moral obligation (*M* = 4.045) and reciprocity (*M* = 4.039) had the highest means, whereas sentimental quality was lower (*M* = 3.455). Overall happiness averaged 3.412 (SD = 0.674); joy of living was highest (*M* = 3.679) and personal fulfillment lowest (*M* = 3.152). Skewness ranged from −0.697 to −0.041 for the happiness scores and from −0.658 to −0.114 for the gratitude scores, indicating limited negative asymmetry overall, with the most pronounced left skew in joy of living and moral obligation. The total gratitude and happiness scores had skewness values of −0.179 and −0.180, respectively, and were therefore descriptively close to symmetric. These coefficients describe asymmetry only: they do not establish that the scores follow normal distributions.

**Table 2:** Reliability and descriptive statistics for gratitude and happiness.

Measure/domain	*k*	*M*	SD	95% CI for *M*	Skew	α (95% bootstrap CI)	ω
Gratitude total	18	3.976	0.438	[3.938, 4.015]	−0.179	0.807 [0.777, 0.831]	0.823
Reciprocity	9	4.039	0.497	[3.996, 4.083]	−0.356	0.770 [0.731, 0.802]	0.786
Moral obligation	7	4.045	0.548	[3.997, 4.092]	−0.658	0.683 [0.633, 0.723]	0.704
Sentimental quality	2	3.455	0.926	[3.374, 3.535]	−0.114	0.628 [0.539, 0.703]	–
Happiness total	27	3.412	0.674	[3.353, 3.471]	−0.180	0.942 [0.933, 0.950]	0.943
Positive sense of life	11	3.525	0.780	[3.457, 3.593]	−0.332	0.902 [0.887, 0.915]	0.904
Life satisfaction	6	3.287	0.771	[3.219, 3.354]	−0.082	0.844 [0.819, 0.864]	0.845
Personal fulfillment	6	3.152	0.759	[3.086, 3.218]	−0.041	0.799 [0.764, 0.828]	0.805
Joy of living	4	3.679	0.827	[3.606, 3.751]	−0.697	0.838 [0.807, 0.866]	0.840

Scores are item-level averages on the original 1–5 metric. Confidence intervals for α used 3,000 bootstrap resamples. Model-based ω was not estimated for the two-item sentimental-quality dimension.

**Figure 1:**
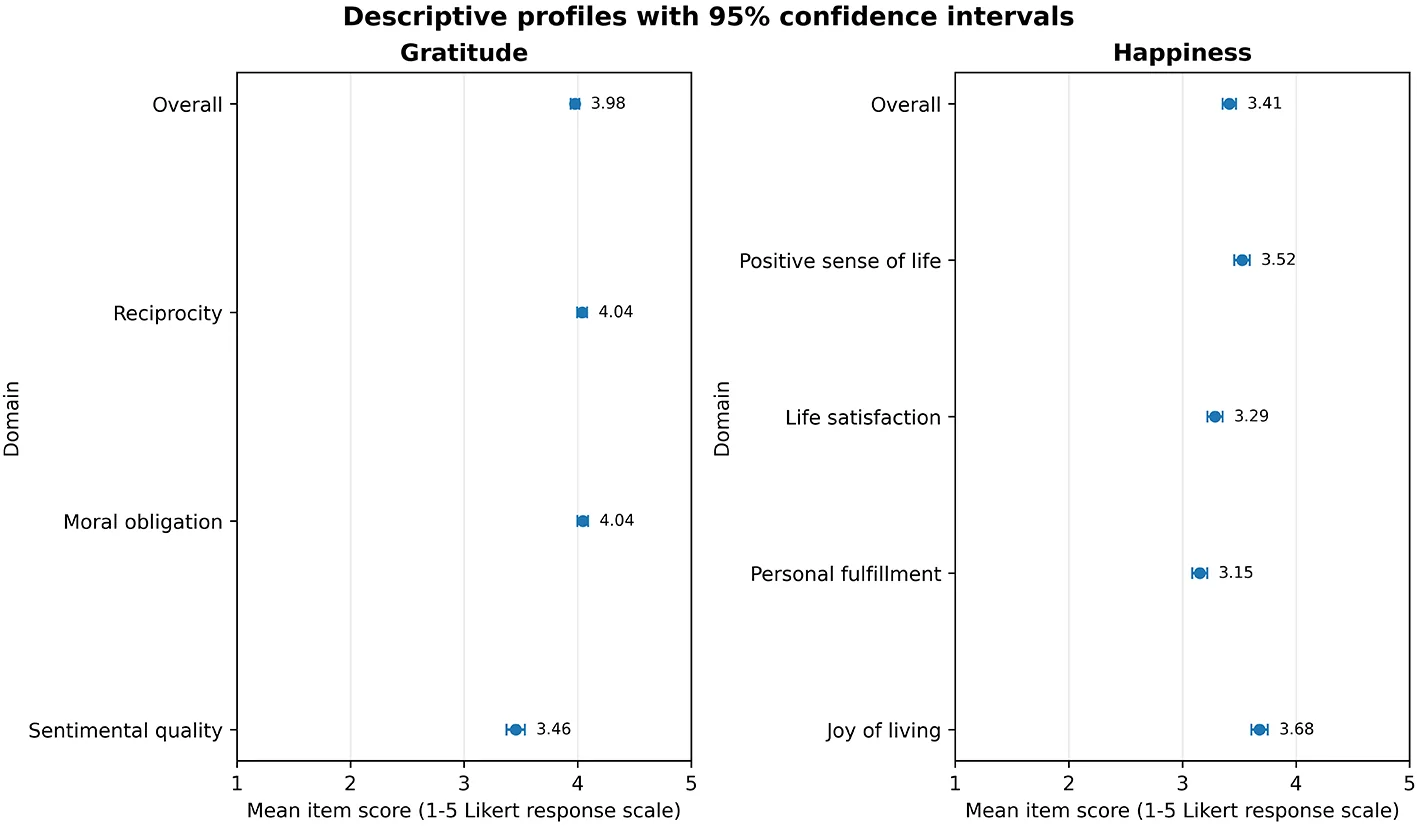
Descriptive profiles of gratitude and happiness domains. Points are mean item scores; horizontal lines are 95% confidence intervals. All scales use the 1–5 response metric; *N* = 507.

### 3.3 Association and robustness analyses

Gratitude was positively associated with overall happiness, ρ = 0.447, 95% bootstrap CI [0.368, 0.519], *p* < 0.001 (Table 3). Positive associations were also observed with positive sense of life, ρ = 0.413, 95% CI [0.331, 0.489]; life satisfaction, ρ = 0.372, 95% CI [0.288, 0.453]; personal fulfillment, ρ = 0.351, 95% CI [0.272, 0.431]; and joy of living, ρ = 0.420, 95% CI [0.341, 0.496]. All five Holm-adjusted *p-*values were < 0.001. The confidence intervals overlapped (Figure 2); no formal comparison of the dependent correlations was conducted, so differences among the four dimension-specific coefficients were not inferred.

**Table 3:** Spearman correlations between gratitude and happiness outcomes.

Outcome	ρ	95% bootstrap CI	*p*	Holm-adjusted *p*	*N*
Overall happiness	0.447	[0.368, 0.519]	< 0.001	< 0.001	507
Positive sense of life	0.413	[0.331, 0.489]	< 0.001	< 0.001	507
Life satisfaction	0.372	[0.288, 0.453]	< 0.001	< 0.001	507
Personal fulfillment	0.351	[0.272, 0.431]	< 0.001	< 0.001	507
Joy of living	0.420	[0.341, 0.496]	< 0.001	< 0.001	507

Confidence intervals used 5,000 paired bootstrap resamples. Holm adjustment controlled the family of five tests.

**Figure 2:**
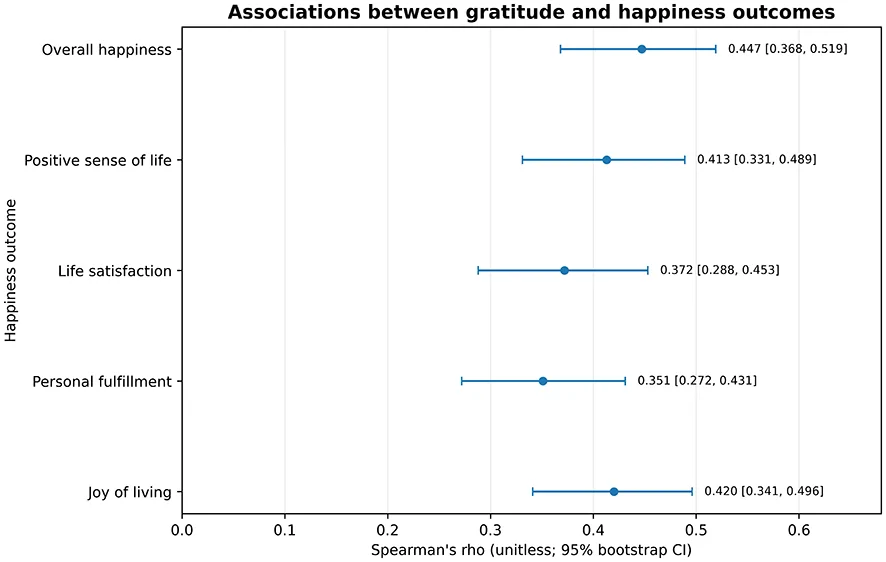
Magnitude and precision of gratitude–happiness associations. Points represent Spearman's ρ; horizontal lines represent 95% bootstrap confidence intervals. *N* = 507 for every estimate.

The supplementary unadjusted linear model also showed a positive association between standardized gratitude and happiness scores, β = 0.457, 95% HC3 CI [0.379, 0.536], *p* < 0.001, *R*^2^ = 0.209. This model explained 20.9% of the observed variance in happiness scores without adjustment for demographic or academic characteristics and therefore does not establish an association independent of potential confounders.

Excluding the participant whose F5 response was reconstructed produced an essentially unchanged estimate (ρ = 0.448, *N* = 506, vs. ρ = 0.447, *N* = 507, with the reconstructed value included). The overall association was therefore insensitive to this single reconstructed response.

## 4 Discussion

The purpose of this study was to determine the relationship between gratitude and happiness, as well as between gratitude and each dimension of happiness, among students at the Professional School of Education of a public university in Tacna.

The central finding indicates a positive, moderately sized association between gratitude and happiness, ρ = 0.447, 95% bootstrap CI [0.368, 0.519]. The supplementary unadjusted linear model was consistent with this result (standardized β = 0.457, 95% HC3 CI [0.379, 0.536]). The association was also stable after excluding the participant with the reconstructed F5 response. This result is consistent with evidence from other university populations: Kalamatianos et al. (2023) reported a stronger relationship between gratitude and subjective happiness (*r* = 0.69) among engineering students, while Rojas-Solís et al. (2022) reviewed happiness research in Latin American university populations. These findings support an association between the constructs, but neither the present design nor its sensitivity analysis establishes causality or rules out confounding.

The difference between the present coefficient and the larger coefficient reported by Kalamatianos et al. (2023) should be interpreted cautiously. The studies differ in disciplinary composition, instruments, and research context, and a Pearson correlation and a Spearman correlation do not estimate precisely the same relationship. Differences in score variability and measurement reliability can also affect their magnitude. Thus, the smaller point estimate in Tacna is not evidence that gratitude is less relevant to prospective teachers than to engineering students. Testing that proposition would require comparable measures and a direct comparison between disciplinary groups.

A plausible interpretation is that students who notice and value support from others also evaluate their lives more positively. In teacher preparation, such support may arise in relationships with peers, lecturers, and school mentors, while meaningful professional goals may provide another connection between gratitude and happiness. Zhang et al. (2022) identified meaning in life and career calling as statistical mediators of associations involving gratitude and career goal self-efficacy in China. Their findings offer a relevant interpretive framework, although the present study did not measure these mediators, perceived support, or professional identity. Reverse directionality is also plausible: happier students may more readily recognize benefits and express gratitude. These explanations remain hypotheses rather than mechanisms demonstrated by the current data.

Regarding the first specific objective, gratitude was moderately related to positive sense of life, ρ = 0.413, 95% bootstrap CI [0.331, 0.489]. This finding suggests that students with a greater disposition toward gratitude also tend to experience less pessimism, uneasiness, and feelings of failure, in line with Zhang, Tang and Yong (2022) description of the role of gratitude in constructing meaning in life.

The association with positive sense of life is relevant to mental health research, but it cannot establish a lower prevalence of depression or anxiety. The Lima Happiness Scale includes negatively worded evaluations of life; it does not provide a clinical assessment. Gustems-Carnicer and Calderón (2013) examined psychological symptoms directly and found different patterns for approach and avoidance coping among teacher education students. This distinction suggests that future studies should assess both positive functioning and distress, and test whether gratitude explains variation beyond coping strategies and current symptoms. It also cautions against interpreting the present association solely as evidence of protection against mental illness.

Regarding the second objective, the relationship between gratitude and life satisfaction was also moderate, ρ = 0.372, 95% bootstrap CI [0.288, 0.453]. This result is consistent with evidence that gratitude contributes to life satisfaction without fully accounting for it (Chauhan et al., 2025; Kerry et al., 2023), and it suggests that other factors, such as perceived progress toward personal goals or institutional support, also influence students' satisfaction with their university lives.

Teacher education research helps explain why gratitude would account for only part of life satisfaction. Huertas-Delgado et al. (2020) found an association between life satisfaction and the value placed on power in Spanish teacher education students, rather than a uniform pattern in which all socially oriented values were linked to greater satisfaction. Although values and gratitude are different constructs, this finding cautions against assuming that prospective teachers share a single motivational profile. In the Tacna sample, perceived progress toward valued goals and the social meaning of reciprocity may be relevant, but these possibilities cannot be separated with the available measures.

The third specific objective yielded the smallest point estimate: gratitude and personal fulfillment, ρ = 0.351, 95% bootstrap CI [0.272, 0.431]. The association remained statistically robust after multiplicity control. Its comparatively lower point estimate may reflect that personal fulfillment is influenced by material conditions and academic progress as well as interpersonal dispositions. This interpretation is descriptive: the confidence intervals overlap, and the dependent correlations were not formally compared.

Finally, gratitude showed a moderately sized association with joy of living, ρ = 0.420, 95% bootstrap CI [0.341, 0.496], the largest dimension-specific point estimate. This result is consistent with the view that gratitude, as a positive emotion, broadens psychological resources and the disposition to enjoy the present (Yang et al., 2023). It also aligns with evidence that brief gratitude interventions improve positive affect and happiness (Kirca et al., 2023), although the present cross-sectional association should not be interpreted as an intervention effect.

Evidence from various educational environments also suggests the conditions under which wellbeing may be enhanced. In the French study by Legrain et al. (2023), cooperative training increased instructional knowledge but autonomy and motivation did not improve equally across training formats, with the learning-focused Jigsaw condition being more advantageous for autonomy than the teaching-focused condition. In Hong Kong, Datu et al. (2023) discovered that the delivery format influenced the outcomes of a multicomponent intervention for prospective preschool teachers. These findings reinforce the current association and suggest that besides individual dispositions learning arrangements and the type of support might be important. However, the effects of the intervention cannot be deduced from the connection seen here. Both studies find no particular reason of better quality of life.

The consistency of findings across these settings supports the relevance of positive psychological resources to teacher preparation, but does not establish a culturally invariant gratitude-happiness relationship. Expectations about reciprocity, the significance of the work of teaching, and the possibilities for social support may vary between educational systems. The use of instruments established in Peru offers contextual grounding for the present study, but does not imply equivalency with measures used elsewhere. Multisite research should test for measurement invariance and analyze the connection by training program and cultural environment before concluding that variations are due to culture or professional specialty.

Several restrictions should be noted. First, the sample was made up exclusively of teacher education students from a public university in Tacna. Recruitment was based on classroom attendance and voluntary involvement, which may have introduced selection bias if those missing or declining varied in thankfulness or contentment. Therefore, the findings characterize the students who participated in this study and should not be taken to represent all the students of the institution, students in other fields, private institutions, other areas or professors in practice. Without a reference group, it is also hard to tell if the observed link is exclusive to teacher preparation or if it represents a more general tendency among university students. Second, the sample was composed of 61.7% of women. The analyzes do not prove demographic representativeness or control for any confounding by age, sex, academic year or program. Third, the cross-sectional architecture does not allow for causal inference and temporal ordering. Fourth, dependence on self-report instruments introduces common-method risk. Finally, the dependability for the two-item sentimental-quality dimension (α = 0.628) and moral responsibility (α = 0.683) was worse than for the overall gratitude scale; consequently, conclusions should focus on total-score findings and be more careful when interpreting dimension-level thankfulness estimations.

There were a number of potentially significant factors that were not examined including practicum experiences, classroom expectations, perceived institutional support, socioeconomic situations, and present mental health symptoms. These factors may influence thankfulness and happiness both, and their contribution cannot be isolated from the correlation shown. Happiness was the outcome measured, hence the consequences for quality of life, mental health, teaching performance, or the wellbeing of future children are indirect. Longitudinal studies that include admission into teacher education, practicum and professional transition might help clarify temporal ordering and discover when thankfulness is most strongly connected with wellbeing.

These findings suggest the consideration of gratitude-related activities as one possible piece of wider assistance for aspiring teachers. Structured reflection on help received or chances to show thanks might be investigated along with mentorship, coping skills and access to relevant support resources. Böke et al. (2024) found, in a quasi-experimental research, improvements in mental health and wellbeing indicators following a stress-management program for preservice teachers, showing the benefit of assessing structured assistance within teacher training. The program was more than an appreciation test. Likewise, the multicomponent interventions of Datu et al. (2023) and Ekwonye et al. (2026) are unable to establish the independent effect of thankfulness. Future experiments in the Peruvian environment should separate gratitude-specific benefits from general program effects, and evaluate if any gains are maintained. The present findings warrant this review, but do not indicate that gratitude exercises would increase happiness or later teaching practice.

## 5 Conclusions

Among 507 teacher education students at a public university in Tacna, Peru, gratitude showed a positive, moderately sized association with overall happiness (ρ = 0.447, 95% bootstrap CI [0.368, 0.519]). Positive associations extended to all four happiness dimensions: positive sense of life, life satisfaction, personal fulfillment, and joy of living. This pattern indicates that gratitude was associated with several aspects of how participants evaluated and experienced their lives.

The study contributes evidence from a Peruvian teacher education setting to research on gratitude and happiness. It documents this relationship among students preparing for educational roles, although whether the association differs from that in other disciplines remains unresolved. Recruitment through classroom attendance and voluntary participation at a single institution limits generalizability. The cross-sectional, unadjusted analyses also leave temporal ordering and potential confounding unresolved.

For teacher education, these findings provide a basis for evaluating gratitude-focused activities as a possible component of broader student support programs. Longitudinal studies across institutions and disciplines should examine whether the association persists over time and after accounting for relevant demographic, academic, and psychosocial factors. Controlled trials are needed to determine whether such activities produce sustained improvements in happiness and offer benefits beyond existing support. The present findings identify an association relevant to student wellbeing; their practical value depends on establishing whether it can inform effective support during teacher preparation.

## Data Availability

The original contributions presented in the study are included in the article. Further inquiries can be directed to the corresponding author.
